# Quantum-electrodynamical birefringence vanishing in a thermal relativistic pair plasma

**DOI:** 10.1038/srep15866

**Published:** 2015-11-02

**Authors:** Y. S. Huang

**Affiliations:** 1 High Power KrF Excimer Laser Laboratory, China Institute of Atomic Energy, 102413, China

## Abstract

Quantum electrodynamical (QED) birefringence in a thermal relativistic pair plasma with the presence of the strong crossed field: 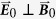, is proposed and investigated. We clarify the coupling relationship and competition between the QED effect and the plasma collective effect and find the critical condition that makes the birefringence vanish. In a relative weak electromagnetic field, the birefringence is dominated by the coupling of the QED-effect, the collective effect and the 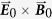 drift effect. In a relative strong electromagnetic field, we obtain the formulations stating the competition between the QED effect and the collective effect and then the critical conditions so that they are canceled with each other and the birefringence vanishes. With our results, a new possible scheme is proposed to estimate the thickness of the magnetosphere in a millisecond pulsar and the plasma density of a pulsar, if the magnetic field is known beforehand.

Nonlinear quantum electrodynamical (QED) effects are of great interest^1^,^2^,^3^ for pair plasmas in astrophysical environments^4^,^5^ and that produced in the laboratory environment with ultra-intense laser pulses^1^,^6^,^7^,^8^,^9^. For circular polarized lasers propagating along a homogenous strong magnetic field in a pair plasma, Marklund and coworkers found new quantum electrodynamical modes^1^,^2^. They also focussed some low-frequency modes applicable in the pulsar environment and numerous nonlinear QED effects^1^. For a low frequency linear-polarized wave propagating perpendicular to a strong magnetic field, Brodin and coworkers^3^ investigated the nonlinear dynamics of QED photon splitting and found a more efficient decay channel of coupled nonlinear electromagnetic waves in magnetized pair plasmas. However, their model was created on the assumption: the electromagnetic wave frequency and the electron(positron) plasma frequency are much smaller than the electron (positron) Larmor frequency. Birefringence is also crucially important and because it may affect the polarization evolution of the linear-polarized wave propagating perpendicular to a strong magnetic field. Lundin^10^ considered the photon propagation in a magnetized background. However, their plasma treatment is only valid for a strong magnetic field (see the conclusion in ref. 10). Mubashar^11^ obtained the linear wave theory in a magnetic quantum plasma, while they did not discuss the influence of collective effects of thermal pair plasmas and the QED effect on birefringence in detail.

The studies on field-induced birefringence are mainly focused on the influence of the quantum vacuum^12^,^13^,^14^,^15^,^16^,^17^,^18^,^19^, the strong non-uniform electric field of gases^20^,^21^,^22^, the spin plasma effect for a strong magnetic field^10^ and the coupling between the QED vacuum effect and thermal plasma effect^23^,^24^,^25^,^26^,^27^,^28^,^29^,^30^ and so on. PVLAS collaboration^16^,^17^, BMV collabration^18^,^22^ and Q & A (quantum electrodynamics and Axion) collaboration^19^ have performed a serials of experiments on vacuum birefringence^16^,^17^,^18^,^19^ (also called Cotton-Mouton effect) and the electric-field-gradient-induced birefringence (EFGB) of gases^20^,^21^,^22^. By improving the detection sensitivity, their experimental results are so close to the theoretical predictions. Both the experiments^22^ and the theories^20^,^21^ show that the Cotton-Mouton constant of EFGB is 7–8 orders of magnitude higher than that in a vacuum with the presence of several Tesla magnetic field. The magnetic field of a pulsar or a magnetized neutron star could reach 10^8−12^ T and induces strong vacuum birefringence of the radiations. In a thermal plasma with presence of a strong slow-varying magnetic field, Pavlov and Shibanov^23^, Ventura, Nagel and M*é*sz*á*ros^24^,^30^, Bulik and Miller^25^, Gnedin, Pavolov and Shibanov^26^, Lai and Ho^27^,^28^, Shannon and Heyl^29^ investigated the radiation polarization evolution due to the QED vacuum effect and the plasma effect of magnetized neutron stars or pulsars. Lai and Ho^31^ suggested the detection of the polarized x-ray as a direct probe of the QED effect. Of particular interest is the so-called “vacuum resonance”, corresponding to the case when the vacuum effect and the plasma effect cancel each other. However, in general, the strong magnetic field and the pulsars are rotating. According to Maxwell equations, a strong rotating electric field perpendicular to the magnetic field exits simultaneously. In the strong crossed field, 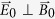, as we known, the pair plasmas in pulsars experience the high-velocity drift, i.e., the 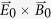 drift, and are relativistic. Therefore, in a thermal relativistic pair plasma with presence of a strong crossed field, the coupling relationship and the competition between the QED effect, the drift effect and the plasma collective effect are necessary to be clarified. Therefore, it is significant to consider the influence of the plasma collective effect on the QED birefringence in a thermal relativistic pair plasma, where the EFGB is nonexistent and the magnetic field is an arbitrary value smaller than the critical Schwinger limit, 4.4 × 10^9^ T.

In this paper, the birefringence in a thermal relativistic pair plasma with the presence of a crossed field, 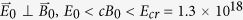 V/m, is investigated, where *E*_*cr*_ is the critical Schwinger field of pair production and c is the light speed in a vacuum. The birefringence is dominated by the coupling of the QED effect, the plasma collective effect and the 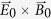 drift effect. The thermal effect is canceled out naturally for wave frequency far larger than the Larmor frequency. We find that the Cotton-Mouton constant in the near-critical-density region is several orders higher than that in a vacuum. In the region of much strong magnetic field and very low plasma density, the competitive relationship between the QED effect and the collective effect is derivated analytically. We find the critical condition that makes the QED effect and the collective effect cancel with each other totally. In the case, the birefringence vanishes. Our results are applicable for probe waves with frequency, which satisfies 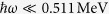, since the effective Lagrangian that they use is only valid well below the pair production threshold. We also discuss applications of our results in pulsar and magnetar atmospheres. Specially, with our results, one can predict the relativistic drift effect in the emission region of interest for millisecond pulsars and normal pulsars.

## Dispersion Relationship

In the following discussions, the parallel polarization corresponds to the probe wave with the electric field parallel to 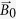 and the perpendicular polarization corresponds to the one with the electric field perpendicular to 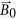.

### Parallel polarization

The dispersion relationship is given by


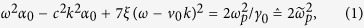


for parallel polarization, where *ω* is the wave frequency, *k* is the wave number, 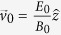 is the 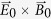 drift velocity, 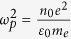, *n*_0_ is the unperturbed plasma density, 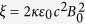, and *ε*_0_, *μ*_0_ are the permittivity and the permeability of vacuum respectively, 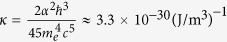, *m*_*e*_ is the electron rest mass, *α* is the fine-structure constant, 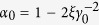 and 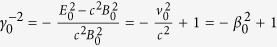.

### Perpendicular polarization

For the perpendicular polarization, it contains an electromagnetic wave: exp[*i*(*k*_*em*_*z* − *ω*_*em*_*t*)], and an electrostatic wave due to the Larmor gyration: exp[*i*(*k*_*s*_*z* − *ω*_*s*_*t*)]. The dispersion relationship of the electromagnetic wave is obtained


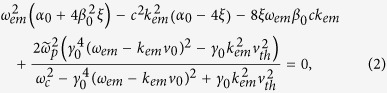


where 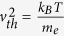 and *k*_*B*_*T* is the plasma temperature. We assume that the electron temperature is equal to the positron temperature.

## QED Birefringence in QED-Pair Plasmas

Birefringence in QED-pair plasmas will be investigated in three cases.

*Case I: In the* 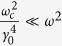 *region*. For the weak field, i.e., the low larmor frequency compared with the probe wave frequency, or for the relativistic drift velocity, *γ*_0_ ≫ 1, it is satisfied: 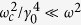. In this case, the dispersion relationship becomes a quadratic equation similar to that of the parallel polarization:


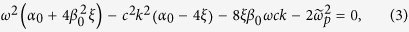


The solutions to the above equation are *n*_*per*,1_ and *n*_*per*,2_, which correspond to the refractive indices of the positive propagating wave and the counter-propagating wave. Assuming 
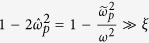
, with the linearization of the refractive indices for parallel polarization and perpendicular polarization, the refractive-index difference is obtained:


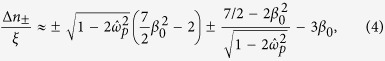


where 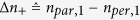 in the positive z direction and 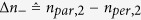 in the negative z direction, *n*_*par*_ represents the refractive indices for parallel polarization.

**Figure 1:**
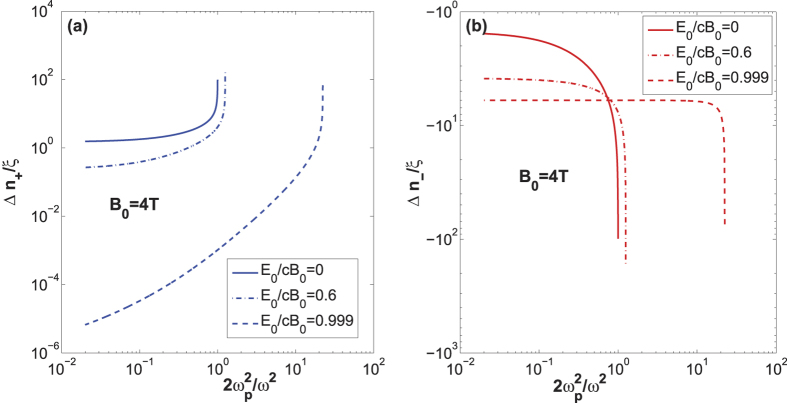
The dependence of the refractive-index difference,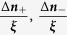 on the pair-plasma density, i.e., the ratio of the plasma frequency to the probe-wave frequency for *B*_0_ = 4 T. (**a**) 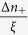 in the positive z direction. It tends to zero as *β*_0_ → 1. (**b**) 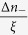 in the negative z direction. It tends to −6 as *β*_0_ → 1.

Equation (4) shows the coupling of the QED effect, *ξ*, the collective effect, *ω*_*p*_ and the drift effect, *β*_0_. For *E*_0_ = 0, *β*_0_ = 0, we get 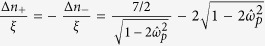, which is 3/2 if *ω*_*p*_ = 0 and is the same as that of vacuum birefringence in a strong homogenous magnetic field. A linear polarization probe wave becomes elliptical polarized with ellipticity 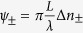 if 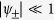, where *L* is the propagating length and *λ* is the wavelength. As shown by the solid line in Fig. 1(a,b), for *E*_0_ = 0, if a proper plasma density is chosen, the ellipticity can be amplified by several orders of magnitude compared with that in a vacuum. Since the Cotton-Mouton constant is 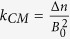, the above discussions on the refractive-indices difference are also correct for the Cotton-Mouton constant.

However, the amplification by the collective effect is canceled partially by the increasing relativistic drift effect as shown by the dash-dot lines and the dashed lines in Fig. 1(a,b). As *β*_0_ tends to the unit, 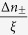 tends to a constant.

For *E*_0 _≈ *cB*_0_, *β*_0_ ≈ 1, we get *γ*_0_ → ∞, 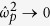 and 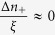 as shown by the dashed line in Fig. 1(a). The QED effect and the drift effect are canceled with each other. 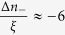 and does not rely on the plasma density or the wave frequency as shown by the dashed line in Fig. 1(b). It is consistent with Eq. (16–17) in ref. 32 and Eq. (15) in ref. 33 in the interaction between a counter-propagating probe wave and a strong low-frequency plane wave.

**Figure 2:**
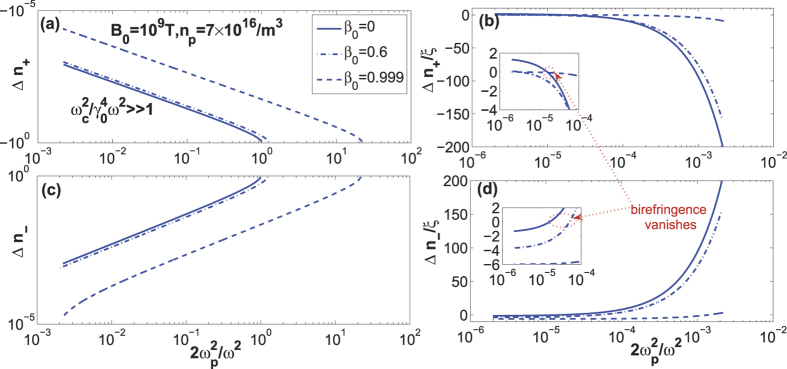
The dependence of the refractive-index difference, 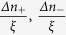 on the ratio of the plasma frequency to the probe-wave frequency for *B*_0_ = 10^9^ T, *n*_*p*_ = 7 × 10^16^/m^3^, *k*_*B*_*T* = 1 keV and a serials of drift velocities, *β*_0_ = 0, 0.6, 0.999. (**a**,**b**) Δ*n*_+_ in the positive z direction. It tends to −1 in the near-critical-density region for the weak-relativistic case; (**c**,**d**) Δ*n*_−_ in the negative z direction. It tends to 1 in the near-critical-density region for the weak-relativistic case. The red dotted line shows the region where the birefringence vanishes.

*Case II: In the* 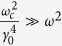 *region*. For the strong magnetic field, i.e., the Larmor frequency far larger than the probe wave frequency, 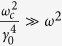 satisfies. In this case, the dispersion relationship is also simplified to be a quadratic equation by neglecting 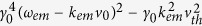 in the denominator of the fourth item of Eq. (2):


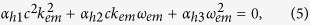


where 
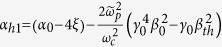
, 
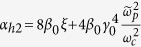
 and 
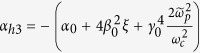
. The term *α*_*h*1_ shows that the influence of the plasma temperature on the refractive index can be neglected. The solutions of the above equation, *n*_*per*,1_ and *n*_*per*,2_ also do not rely on the wave frequency. Set 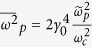, we have 
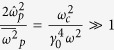
. The undamped wave propagating in the pair plasma requires 
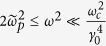
. Therefore, we have 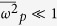. *α*_*h*2_ and *α*_*h*3_ contain the couping terms, *β*_0_*ξ* and 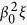, between the QED effect and the drift effect. The couping between the collective effect and the drift effect also exists and competes with the couping between the QED effect and the drift effect. In a rare plasma, the collective effect can be neglected and the coupling of the QED effect and the drift effect dominates for the perpendicular polarization. With *n*_*per*,1_ and *n*_*per*,2_ and the refractive indices for the parallel polarization, the refractive-index differences have been calculated and shown in Fig. 2(a–d). In the region: 
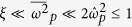
 or 
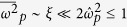
, where the relative plasma density is much larger than the QED parameter, *ξ*, the QED effect is overwhelmed by the collective effect and is shown in Fig. 2(a,c). However, in the region: 
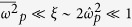
, where the relative plasma density is much small and comparable with *ξ*, the refractive-index difference is given by:


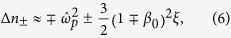


by the linearization of the refractive indices. Figure 2(b,d) show the accurate calculation results of the refractive indices in a rare plasma. They are consistent with the linearization results, Eq. (6). They clearly show the competition between the QED effect and the collective effect in a rare plasma. The influence of the drift effect is also indicated. From that, the critical plasma density, for which the QED effect and the collective effect are canceled with each other, satisfy 
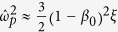
 and 
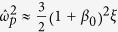
 for the forward-propagating wave and counter-propagating wave, respectively. In that case, the birefringence vanishes.

For 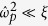 and *β*_0_ = 0, 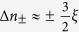, which are consistent with that of the vacuum birefringence^13^,^14^,^15^. As shown in Fig. 2(b,d), the difference of the refractive indices tend to 3*ξ*/2 and −3*ξ*/2 for the forward-propagating wave and counter-propagating wave as 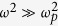 and *β*_0_ = 0, respectively. And they tend to 0 and −6*ξ* for the relativistic drift velocity, respectively. The drift effect and the QED effect dominate in this case.

**Figure 3:**
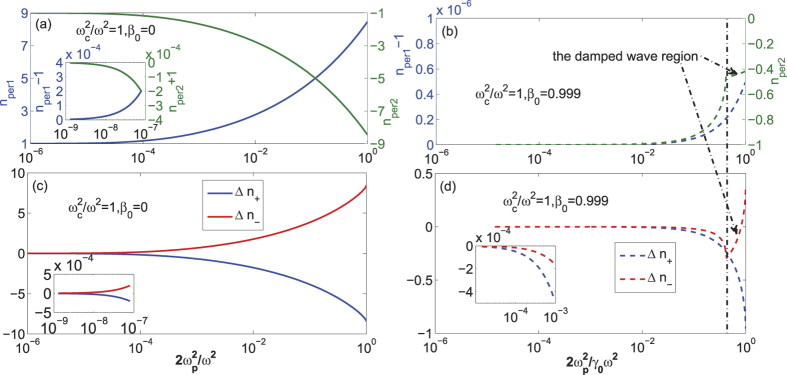
(**a**,**b**) The dependence of the refractive indices on 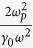 for perpendicular polarization and 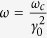; (**c**,**d**) the dependence of the the refractive-index difference, Δ*n*_+_, Δ*n*_−_ on 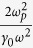. *k*_*B*_*T* = 100 eV and *B*_0_ = 4 T. In (**a**,**c**), *β*_0_ = 0. In (**b**,**d**), *β*_0_ = 0.999.

*Case III:* 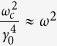. In this case, the dispersion relationship, i.e., Eq. (2) needs to be solved. Here two typical cases are considered: low *B*_0_ of several Tesla and high *B*_0_ of 10^9^ T. For low *B*_0_ of about several Tesla, the wave frequency is about 10^12^ rad/s and the wavelength is about several millimeters. The plasma densities are chosen to allow the undamped waves propagate. For example, the plasma density should be smaller than 7.76 × 10^19^/m^3^ for *B*_0_ = 4 T and *β*_0_ = 0. Figure 3 shows the refractive indices and the refractive-index difference *vs* 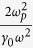. In the relativistic case, the wave is damped before 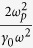 comes to the unit as shown by the damped wave region in Fig. 3(b,d). Since *ξ* = 8.4 × 10^−23^ for *B*_0_ = 4 T, the influence of the quantum-vacuum polarization is overwhelmed by the collective effect and drift effect totally in this case.

For high *B*_0_ of about 10^9^ T, the corresponding wave frequencies are about 1.75 × 10^20^ rad/s and 3.5 × 10^17^ rad/s for *β*_0_ = 0 and *β*_0_ = 0.999 respectively. The corresponding wavelengthes are about 0.01 nm and 5 nm respectively, which is in the x-ray region. In this case, the critical plasma density is so high to reach generally. For a relative low plasma density, 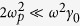, the refractive-index differences of the forward propagating wave are 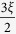 and zero for *β*_0_ = 0 and *β*_0_ = 0.999 respectively. The ones of the counter-propagating wave are 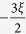 and −6*ξ* respectively. The results are the same as that of the vacuum birefringence. The QED effect and the drift effect dominate, while the collective effect is overwhelmed.

## Applications and Discussions

For 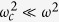 and *E*_0_ = 0, in the near-critical-density region, i.e., 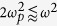, the Cotton-Mouton constant, i.e., the refractive-indices difference, might be several orders of magnitude larger than that of the vacuum birefringence. For example, |Δ*n*_±_| ≈ 35 *ξ* for 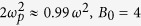 T and *β*_0_ = 0. It proposes a feasible scheme to detect birefringence due to the coupling of the QED effect and the collective effect precisely. However, if the plasma density is slightly larger than the critical density, the probe wave will be damped and exhausted quickly. Therefore, the precise control of the plasma density is required.

In the magnetosphere of pulsars and magnetars, strong linear polarization radiations mainly come from curvature radiations, synchrotron radiations, or inverse Compton scatters of energetic electrons in the inner gap and are emitted along the tangent of the magnetic lines. Some of the waves experience magnetic field components perpendicular to the tangent direction when they propagate in the magnetosphere. With the GL model^4^, the perpendicular magnetic-field components are about 10^5~8^ T for a ‘normal’ pulsar and 10^1~4^ T for a millisecond pulsar respectively and increase the angle between the emitted direction of the waves and the magnetic-field direction. The QED birefringence of the waves exists and changes the linear polarization to the elliptical polarization. With reference to the discussions in refs 3, 4, 5 and references therein, the typical pair plasma densities satisfy *n*_*p*_ = 7 × 10^16^/m^3^ for a ‘normal’ pulsar and *n*_*p*_ = 7 × 10^12^/m^3^ for a millisecond pulsar. Assuming the thickness of the magnetosphere is about 1000 km, with 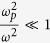 and 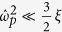, the birefringence of a x-ray or a gamma ray from a ‘normal’ pulsar is dominated by the QED effect and the ellipticity is not a small quantity and could reach any possible value from zero to 
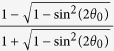
, where *θ*_0_ is the initial angle of the polarization vector with respect to the perpendicular magnetic-field component. With Eq. (6), the critical wavelength that makes birefringence vanish is about 355 nm. Therefore, for visible lights or RF waves, the birefringence is dominated by the plasma collective effect and the ellipticity could also reach 
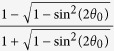
. For 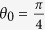, 
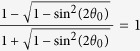
 and the elliptical polarization becomes the circle polarization. Therefore, it is infeasible to deduct the magnetic field or the thickness of the magnetosphere of a ‘normal’ pulsar directly from the ellipticity. However, the ellipticity of a high-frequency wave with wavelength smaller than 3 μm from a millisecond pulsar is dominated by the QED effect and is of the order of 10^−8^ ~ 10^−1^. Therefore, the thickness of the magnetosphere of a millisecond pulsar could be estimated by


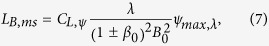


where *ψ*_*max*,*λ*_ is the maximum ellipticity of the wave with the wavelength of *λ* ≪ 3 μm, and 
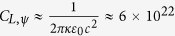
. In the nonrelativistic case, i.e. *β*_0_ = 0, Eq. (7) is simplified to be 
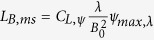
.

However, with the prediction of the birefringence vanished by the competition between the QED effect and the collective effect, the wave with the critical wavelength governed by Equation (6) is linear polarized or is approximately linear polarized and could be identificated from the radiations. If the dependence of the degree of elliptical polarization on the wave frequency or the wavelength is obtained, the degree of elliptical polarization should achieve a minimum value at the critical wavelength. Assume the critical wavelength is *λ*_*c*,*l*_ and the critical wave frequency is *ω*_*c*,*l*_. With Eq. (6), the relationship between the plasma density and the magnetic field is given by


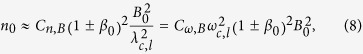


where 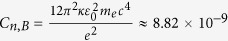 and 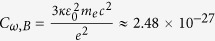. Therefore, with the obtained magnetic field and the critical wavelength, the plasma density could be estimated. In the nonrelativistic case, *β*_0_ = 0, Eq. (8) is simplified to be 
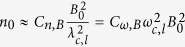
, corresponding to the plasma mass density 
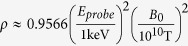
, which is consistent well with the “vacuum resonance” condition, Eq. (1.1) in ref. 27, where *E*_*probe*_ stands for the photon energy.

As discussed by M*é*z*á*ros^30^, and Lai and Ho^27^,^34^, Faraday depolarization may affect the polarization evolution of the radiation from pulsars or magnetized neutron stars. The details of the discussions on Faraday depolarization beyond the range of this paper, since the dispersion relationship of an electromagnetic wave propagating along the strong magnetic field in a thermal relativistic pair plasma is needed. However, fortunately, Lai and Ho concluded that the breakdown of Faraday depolarization occurs near the “vacuum resonance”^34^. Therefore, with the vanishing of the QED birefringence, i.e., Eq. (8), our proposal to detect the plasma density or the magnetic field of a pulsar is still effective.

In a cold plasma with the presence of a strong magnetic field, Shannon and Heyl^29^ numerically and detailedly calculated the phase-averaged polarization induced by the magnetospheric birefringence, i.e., the coupling between the QED effect and the plasma effect in the atmosphere of a neutron star. Their calculation results partially validates that the vanishing of QED birefringence or the “vacuum resonance” is a considerable phenomena to diagnose parameters of plasmas or magnetosphere in pulsars or magnetized neutron stars. Furthermore, considering the relativistic effect and the 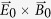 drift effect, the vanishing condition of QED birefringence obtained by our model is more appropriate for rotating pulsars or rotating millisecond pulsars.

With our results, it is important to discuss the relativistic drift effect on the QED birefringence vanishing in the emission region of interest for pulsars. For a millisecond pulsar, *ω*_*c*_ = 10^12~15^ Hz. Therefore, if the QED birefringence vanishing appears for a radio-frequency wave or a visible wave, 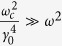 is required. Therefore, *β*_0_ ≪ 1, i.e., *γ*_0_ ≈ 1, is required. For a normal pulsar, *ω*_*c*_ = 10^16~19^ Hz, *n*_*p*_ = 7 × 10^16^/m^3^ and *ω*_*p*_ = 1.5 × 10^10^ Hz. Analogously, if the QED birefringence vanishing appears for a visible wave with the frequency of about 10^14~15^ Hz or a soft x-ray, *γ*_0_ ≈ 1 is also required. If the pair plasma is strong relativistic, i.e., *β*_0_ ≈ 1 and *γ*_0_ ≫ 1, the QED birefringence vanishing can appear for a radio-frequency wave from a normal pulsar, since 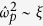 should be satisfied simultaneously.

In conclusion, our results are obtained in relativistic pair plasmas. With the results, a possible new scheme was proposed to estimate the thickness of the magnetosphere in a millisecond pulsar and the plasma density of a pulsar or magnetar. As an important result, the expected value of *β*_0_, i.e. the velocity of the guiding center, is estimated and is small for millisecond pulsars and normal pulsars for the emission regions of interest. Considering only the collective effect and assuming the ions uniform and cold and 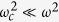, the dispersion relationships and the main results about the coupling relationship and competition in a general relativistic plasma can be obtained from Eqs. (1), (3), (4) and (6) by replacing 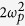 by 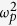. In a general underdense plasma with the presence of relative-weak magnetic field of about several Tesla, rather than the collective effect, the contributions from the EFGB of gases are dominant^20^,^22^. However, the EFGB is overwhelmed by the QED birefringence for strong magnetic fields of the order of 10^3~9^ T. Our results are valuable references in the studies of other nonlinear QED effects in relativistic plasmas.

## Methods

First, we assume the strong classical field as: 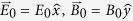, where *E*_0_ < *cB*_0_ < *E*_*cr*_, *c* is the light speed in vacuum, 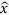 and 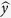 are the unit vector of the x, y direction respectively. Let 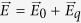 and 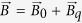 be the total electromagnetic field, where *E*_*q*_, *B*_*q*_ are the nonclassical part with expressions: 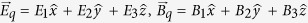. Therefore, *E*_2_, *B*_2_ are the parts parallel to the external magnetic field and *E*_1_, *B*_1_, *E*_3_, *B*_3_ are the parts perpendicular to 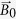. Let 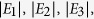 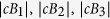 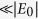. The wave is along the z direction with 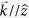.

Using the effective Lagrangian 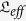
1, the QED corrected electric displacement field 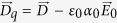 and the magnetic field strength 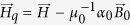 are given by: 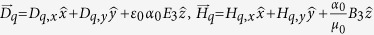, where 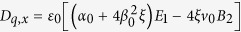, *D*_*q*,*y*_ = *ε*_0_[(*α*_0_ + 7*ξ*)*E*_2_ + 7*ξv*_0_*B*_1_], 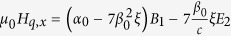, 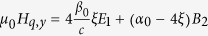. For *E*_0_ = *cB*_0_, we have *α*_0_ = 1.

It is the starting point that the QED-corrected Maxwell equations^1^ with the plasma current: 
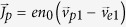
, where *n*_0_ is the undisturbed plasma density and 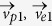 are the disturbed velocity of positron and electron, respectively. We assume that the temperature of the electrons and positrons the same as *k*_*B*_*T*_*e*_ = *k*_*B*_*T*_*p*_ = *k*_*B*_*T*, where *k*_*B*_ is the Boltzmann constant, *n*_*p*_ and *n*_*e*_ represent electron density and positron density respectively. They are governed by the following relativistic momentum equations:


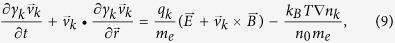


where the relativistic factor 
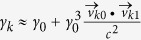
, the subscript _*k*_ represent _*p*_ and _*e*_ for positron and electron respectively, and *q*_*p*_ = *e*, *q*_*e*_ = −*e*. Here, the linearization of *γ*_*k*_ requires that 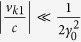. Therefore, the field magnitude of the perturbed wave should be small and satisfies: 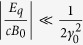.

We make the wave assumptions: 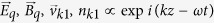. Then the linearization of the corrected Maxwell equations and the momentum equations of electrons and positrons can be obtained easily and we get *B*_3_ = 0 and *en*_0_(*v*_*p*1*z*_ − *v*_*e*1*z*_) = *iωα*_0_*ε*_0_*E*_3_. The zero-order items of the momentum equation give: 
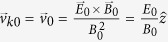
, which characterizes the 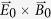 drift. Combining the linearization of the corrected Maxwell equations and the momentum equations of electrons and positrons, the dispersion relationship is obtained after some straightforward algebra. For the perpendicular polarization, it contains an electromagnetic wave: 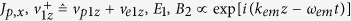, and an electrostatic wave due to the Larmor gyration: 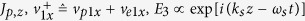.

## Additional Information

**How to cite this article**: Huang, Y. S. Quantum-electrodynamical birefringence vanishing in a thermal relativistic pair plasma. *Sci. Rep.* **5**, 15866; doi: 10.1038/srep15866 (2015).

## Supplementary Material

Supplementary Information
