# Supplementary material for: Quantum-electrodynamical birefringence vanishing in a thermal relativistic pair plasma
Source: Sci Rep. 2015 Nov 2;5:15866. doi: 10.1038/srep15866 (PMC4629147; doi:10.1038/srep15866)
Supplement: Supplementary Information [file srep15866-s1.pdf]

Supplementary Material：Quantum-electrodynamical birefringence vanishing in a thermal relativistic pair plasma

Y. S. Huang

- **The derivation of the dispersion relationships**

The starting point is the effective Lagrangian：

$$\mathcal{L}_{\mathrm{eff}} = \epsilon_0 \frac{E^2 - c^2B^2}{2} + \kappa\epsilon_0^2\left[(E^2 - c^2B^2)^2 + 7c^2\left(\vec{E}\cdot\vec{B}\right)^2\right], \qquad \text{(supp. 1)}$$

where $\vec{E}$ and $\vec{B}$ is the total electric and magnetic field respectively.

Then the linearization of the corrected Maxwell equations are given by:

$$\omega\tilde{B}_1 = -k\tilde{E}_2$$
$$\omega\,\tilde{B}_2 = k\tilde{E}_1$$
$$\frac{ik}{\mu_0}\left[(\alpha_0 - 7\beta_0^2\xi)\tilde{B}_1 - \frac{7\xi\beta_0}{c}\tilde{E}_2\right] = en_0(v_{p1y} - v_{e1y}) - i\omega\epsilon_0\left[(\alpha_0 + 7\xi)\tilde{E}_2 + 7\xi\, v_0\tilde{B}_1\right] \qquad \text{(supp. 2)}$$
$$-\frac{ik}{\mu_0}\left[(\alpha_0 - 4\xi)\tilde{B}_2 + 4\xi\frac{\beta_0}{c}\tilde{E}_1\right] = en_0(v_{p1x} - v_{e1x}) - i\omega\epsilon_0[(\alpha_0 + 4\xi\beta_0^2)\tilde{E}_1 - 4\xi\, v_0\tilde{B}_2],$$

In order to linear the momentum equations, we need the following facts:

$$\gamma_k\,\vec{v}_k = \gamma_0\vec{v}_{k0} + \gamma_0\vec{v}_{k1} + \gamma_{k1}\vec{v}_{k0},$$
$$\gamma_k \approx \gamma_0 + \gamma_0^3\vec{v}_{k0}\cdot\frac{\vec{v}_{k1}}{c^2} \qquad \text{(supp. 3)}$$

And

$$\vec{E} = \vec{E}_0 + \vec{E}_q,$$
$$\vec{v}_k \times \vec{B} = \vec{v}_{k0} \times \vec{B}_0 + \vec{v}_{k1} \times \vec{B}_0 + \vec{v}_0 \times \vec{B}_q \qquad \text{(supp. 4)}$$

Therefore, the zero-order items of the momentum equation give:

$$\vec{v}_{k0} = \vec{v}_0 = \vec{E}_0 \times \frac{\vec{B}_0}{B_0^2} = \frac{E_0}{B_0}\hat{z}$$

which characterizes the $\vec{E}_0 \times \vec{B}_0$ drift.

By linearing of the momentum equations of electrons and positrons and after some algebra, we can get:

$$i\gamma_0(kv_0 - \omega)\frac{J_{p,x}}{n_0e} = \frac{2e}{m_e}(E_1 - v_0B_2) - \omega_c v_{1z}^{+}, \qquad \text{(supp. 5)}$$

$$v_{1z}^{+}=\frac{i\omega_c(\omega-kv_0)}{\gamma_0^3(kv_0-\omega)^2-k^2v_{th}^2}\frac{J_{p,x}}{n_0e},$$

and

$$v_{1x}^{+}=-\frac{\omega_c}{i\gamma_0(kv_0-\omega)}\frac{J_{p,z}}{n_0e},$$

$$\frac{i(\gamma_0^3(kv_0-\omega)^2-k^2v_{th}^2)}{kv_0-\omega}\frac{J_{p,z}}{n_0e}=\frac{2e}{m_e}E_3+\omega_c v_{1x}^{+}, \qquad \text{(supp. 6)}$$

And

$$v_{k1y}=\frac{1}{i\gamma_0(kv_0-\omega)}\frac{q_k}{m_e}(E_2+v_0B_1), \qquad \text{(supp. 7)}$$

Combing Eq. (supp.7) and the first and the third equation of Eq. (supp. 2), the dispersion relationship of the electromagnetic wave for the parallel polarization can be obtained.

Combing Eq. (supp.5) and the second and the fourth equation of Eq. (supp. 2), the dispersion relationship of the electromagnetic wave for the perpendicular polarization can be obtained.

Combing Eq. (supp. 6) and the relationship: $en_0(v_{p1z}-v_{e1z})=i\omega\alpha_0\epsilon_0E_3$, the dispersion relationship of the electrostatic wave is obtained:

$$\omega_c^2-\gamma_0^4(k_sv_0-\omega_s)^2+\gamma_0k_s^2v_{th}^2=\gamma_0^2(k_sv_0-\omega_s)\frac{2\widetilde{\omega}_p^2}{\alpha_0\omega_s}, \qquad \text{(supp. 8)}$$

- **The derivation process of Eqs. (5).**

The roots of Eq. (2) are:

$$n_{par,1,2}=\frac{-7\beta_0\xi\pm\sqrt{\frac{\Delta}{4}}}{\alpha_0-7\beta_0^2\xi}, \qquad \text{(supp. 9)}$$

where

$$\Delta=4\left[\alpha_0^2-\left(2\widehat{\omega}_p^2-7\gamma_0^{-2}\xi\right)\alpha_0+14\widehat{\omega}_p^2\beta_0^2\xi\right], \qquad \text{(supp. 10)}$$

Since $1-2\widehat{\omega}_p^2\gg\xi$, $\sqrt{\frac{\Delta}{4}}$ can be simplified:

$$\sqrt{\frac{\Delta}{4}}\approx\sqrt{1-2\widehat{\omega}_p^2-4(1-\beta_0^2)\xi+7\gamma_0^{-2}\xi+4\widehat{\omega}_p^2(1-\beta_0^2)\xi+14\widehat{\omega}_p^2\beta_0^2\xi}$$

$$=\sqrt{1-2\widehat{\omega}_p^2}\sqrt{1+\frac{3(1-\beta_0^2)\xi}{1-2\widehat{\omega}_p^2}+\frac{2\widehat{\omega}_p^2(5\beta_0^2+2)\xi}{1-2\widehat{\omega}_p^2}} \qquad \text{(supp. 11)}$$

$$\approx \sqrt{1-2\widehat{\omega}_p^2}\left(1+\frac{\frac{3}{2}(1-\beta_0^2)\xi}{1-2\widehat{\omega}_p^2}+\frac{\widehat{\omega}_p^2(5\beta_0^2+2)\xi}{1-2\widehat{\omega}_p^2}\right)$$

$$= \sqrt{1-2\widehat{\omega}_p^2}+\frac{\frac{3}{2}(1-\beta_0^2)\xi}{\sqrt{1-2\widehat{\omega}_p^2}}+\frac{\widehat{\omega}_p^2(5\beta_0^2+2)\xi}{\sqrt{1-2\widehat{\omega}_p^2}}$$

Therefore the refractive index for the parallel polarization is linearized to be:

$$n_{par,1} \approx \left(-7\beta_0\xi+\sqrt{1-2\widehat{\omega}_p^2}+\frac{\frac{3}{2}(1-\beta_0^2)\xi}{\sqrt{1-2\widehat{\omega}_p^2}}+\frac{\widehat{\omega}_p^2(5\beta_0^2+2)\xi}{\sqrt{1-2\widehat{\omega}_p^2}}\right)(1+(2+5\beta_0^2)\xi)$$

$$= -7\beta_0\xi+\sqrt{1-2\widehat{\omega}_p^2}+\frac{\frac{3}{2}(1-\beta_0^2)\xi}{\sqrt{1-2\widehat{\omega}_p^2}}+\frac{\widehat{\omega}_p^2(5\beta_0^2+2)\xi}{\sqrt{1-2\widehat{\omega}_p^2}}+\sqrt{1-2\widehat{\omega}_p^2}(2+5\beta_0^2)\xi \qquad \text{(supp. 12)}$$

$$= \sqrt{1-2\widehat{\omega}_p^2}+\frac{\frac{7}{2}(1+\beta_0^2)\xi-\widehat{\omega}_p^2(5\beta_0^2+2)\xi}{\sqrt{1-2\widehat{\omega}_p^2}}-7\beta_0\xi$$

With the same derivation, we get:

$$n_{par,2} \approx -\sqrt{1-2\widehat{\omega}_p^2}-\frac{\frac{7}{2}(1+\beta_0^2)\xi-\widehat{\omega}_p^2(5\beta_0^2+2)\xi}{\sqrt{1-2\widehat{\omega}_p^2}}-7\beta_0\xi \qquad \text{(supp. 13)}$$

In the case I, in the $\frac{\omega_c^2}{\gamma_0^4} \ll \omega^2$ region, the refractive index for the perpendicular polarization can also be simplified to be:

$$n_{per,1} \approx \sqrt{1-2\widehat{\omega}_p^2}+\frac{2(1+\beta_0^2)\xi-\widehat{\omega}_p^2(6-2\beta_0^2)\xi}{\sqrt{1-2\widehat{\omega}_p^2}}-4\beta_0\xi$$

$$n_{per,2} \approx -\sqrt{1-2\widehat{\omega}_p^2}-\frac{2(1+\beta_0^2)\xi-\widehat{\omega}_p^2(6-2\beta_0^2)\xi}{\sqrt{1-2\widehat{\omega}_p^2}}-4\beta_0\xi \qquad \text{(supp. 14)}$$

Therefore, Eq. (5) is achieved.

- **The derivation process of Eqs. (7).**

In the case II, the refractive indices for the perpendicular polarization are the solutions

of Eq. (6). With $\overline{\omega^2}_p \ll \xi \sim 2\widehat{\omega}_p^2 \ll 1$, they are linearized to be:

$$n_{per,1} \approx 1 - 4\beta_0\xi - 4\gamma_0^{-2}\xi + 2(3-\beta_0^2)\xi = 1 + 2(1-\beta_0)^2\xi$$
$$n_{per,2} \approx -1 - 4\beta_0\xi + 4\gamma_0^{-2}\xi - 2(3-\beta_0^2)\xi = -1 - 2(1+\beta_0)^2\xi \qquad \text{(supp. 15)}$$

At the same time, the refractive indices for the parallel polarization can be simplified to be:

$$n_{par,1} \approx 1 - \widehat{\omega}_p^2 + \frac{7}{2}(1+\beta_0^2)\xi - 7\beta_0\xi \qquad \text{(supp. 16)}$$

And

$$n_{par,2} \approx -1 + \widehat{\omega}_p^2 - \frac{7}{2}(1+\beta_0^2)\xi - 7\beta_0\xi \qquad \text{(supp. 17)}$$

With Eqs. (supp. 16-17) and (supp. 15), Eq. (7) is achieved.

- **The estimation of the magnetic field experienced by the emission waves in the magnetosphere of a pulsar.**

With reference to the GJ model, the radiations are emitted along the tangent of the magnetic lines. Therefore, the magnetic field experienced by the wave is approximately parallel with the wave direction. Take the conservative estimations: the angle between the wave vector and the magnetic field is about $0.1^o = \frac{0.1}{360} \times 2\pi \approx \frac{1}{1000}$, the thickness of the magnetosphere is 100km. Then the perpendicular component of the magnetic field is about $\frac{1}{1000}B_0 = 10^5$T. As discussed in the "Applications and discussions", the ellipticity due to the QED birefringence could be any value from 0 to $\frac{1-\sqrt{1-\sin^2 2\theta_0}}{1+\sqrt{1-\sin^2 2\theta_0}}$ since the phase difference between the parallel polarization wave and the perpendicular polarization wave could be any value from 0 to 2pi. If the angle is larger than 0.1^o, the thickness of the magnetosphere is larger than 100km, and the field is larger than 10^5T, the results of the ellipticity are the same. Therefore, our estimations of the parameters are conservative, but it will not change the results: it is infeasible to deduct the magnetic field, the thickness of the magnetosphere or plasma density of a 'normal' pulsar directly from the ellipticity.
